# Brain ventricle parcellation using a deep neural network: Application to patients with ventriculomegaly

**DOI:** 10.1016/j.nicl.2019.101871

**Published:** 2019-05-24

**Authors:** Muhan Shao, Shuo Han, Aaron Carass, Xiang Li, Ari M. Blitz, Jaehoon Shin, Jerry L. Prince, Lotta M. Ellingsen

**Affiliations:** a Department of Electrical and Computer Engineering, The Johns Hopkins University, Baltimore, MD 21218, USA; b Department of Biomedical Engineering, The Johns Hopkins University School of Medicine, Baltimore, MD 21205, USA; c Laboratory of Behavioral Neuroscience, National Institute on Aging, National Institutes of Health, Baltimore, MD 20892, USA; d Department of Computer Science, The Johns Hopkins University, Baltimore, MD 21218, USA; e Department of Radiology and Radiological Science, The Johns Hopkins University School of Medicine, Baltimore, MD 21287, USA; f Department of Radiology, University of California San Francisco, San Francisco, CA 94117, USA; g Department of Electrical and Computer Engineering, University of Iceland, Reykjavik, Iceland

**Keywords:** Magnetic resonance imaging, Enlarged brain ventricles, Convolutional neural networks, Labeling, Normal pressure hydrocephalus, Ventricular system

## Abstract

Numerous brain disorders are associated with ventriculomegaly, including both neuro-degenerative diseases and cerebrospinal fluid disorders. Detailed evaluation of the ventricular system is important for these conditions to help understand the pathogenesis of ventricular enlargement and elucidate novel patterns of ventriculomegaly that can be associated with different diseases. One such disease is normal pressure hydrocephalus (NPH), a chronic form of hydrocephalus in older adults that causes dementia. Automatic parcellation of the ventricular system into its sub-compartments in patients with ventriculomegaly is quite challenging due to the large variation of the ventricle shape and size. Conventional brain labeling methods are time-consuming and often fail to identify the boundaries of the enlarged ventricles. We propose a modified 3D U-Net method to perform accurate ventricular parcellation, even with grossly enlarged ventricles, from magnetic resonance images (MRIs). We validated our method on a data set of healthy controls as well as a cohort of 95 patients with NPH with mild to severe ventriculomegaly and compared with several state-of-the-art segmentation methods. On the healthy data set, the proposed network achieved mean Dice similarity coefficient (DSC) of 0.895 ± 0.03 for the ventricular system. On the NPH data set, we achieved mean DSC of 0.973 ± 0.02, which is significantly (*p* < 0.005) higher than four state-of-the-art segmentation methods we compared with. Furthermore, the typical processing time on CPU-base implementation of the proposed method is 2 min, which is much lower than the several hours required by the other methods. Results indicate that our method provides: 1) highly robust parcellation of the ventricular system that is comparable in accuracy to state-of-the-art methods on healthy controls; 2) greater robustness and significantly more accurate results on cases of ventricular enlargement; and 3) a tool that enables computation of novel imaging biomarkers for dilated ventricular spaces that characterize the ventricular system.

## Highlights

• The first deep learning method specifically developed to parcellate the ventricular system of the human brain.
• Presenting results on both healthy controls and normal pressure hydrocephalus (NPH) patients.
• Achieved comparable results with state-of-the-art brain segmentation methods on healthy controls.
• Achieved greater robustness and signicantly more accurate results when segmenting NPH subjects.

## 1 Introduction

**Fig. 1:**
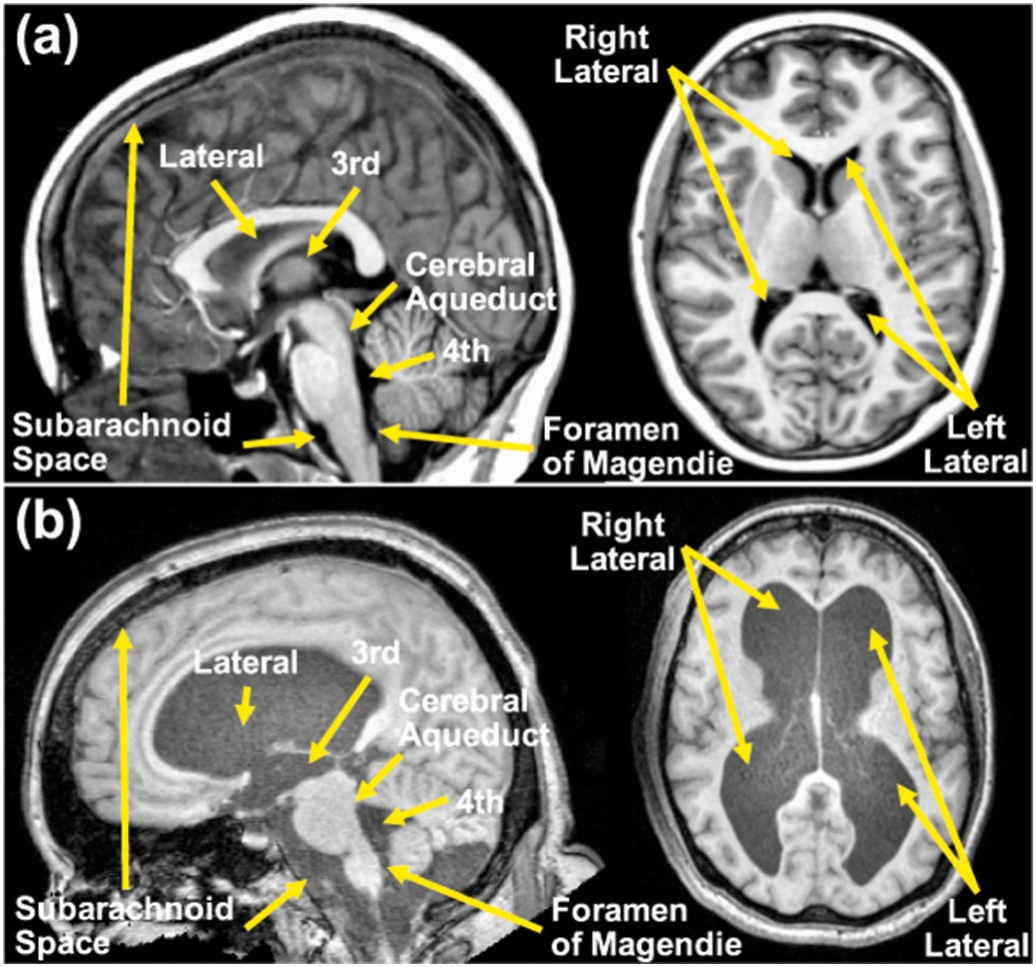
The ventricular system on an MPRAGE T1-weighted MRI of (a): a healthy subject and (b) an NPH subject.

The ventricular system of the human brain consists of four cavities: two large lateral ventricles and the third and fourth ventricles (see Fig. 1(a)). All four ventricles contain strands of a highly convoluted and vascular membranous material called choroid plexus, which is a network of ependymal cells involved in the production of cerebrospinal fluid (CSF). The CSF fills the ventricular space within the brain and bathes the entire central nervous system. The entire volume of CSF is renewed two to three times per day (Nolte, 2009; Bradley, 2015) and disruption of the flow can cause excess CSF to build up leading to the clinical condition called hydrocephalus. In hydrocephalus, the ventricles expand and press against nearby brain tissue causing the brain shape to become distorted (see Fig. 1(b)), leading eventually to brain damage. If this happens over a long period of time, the clinical syndrome of normal pressure hydrocephalus (NPH) may result. In conjunction with ventriculomegaly, symptoms of NPH include cognitive impairment, gait dysfunction, and urinary incontinence (McGirt et al., 2005).

NPH most often affects older adults and may account for more than 5% of all dementia diagnoses (Olivero, 2015). However, unlike other well-known causes of dementia, such as Parkinson's and Alzheimer's disease, once diagnosed, NPH patients have the option of shunt surgery or endoscopic third ventriculostomy (ETV), making the dementia-like symptoms, as well as other symptoms of the disease, potentially reversible. Recent studies suggest that the prevalence of NPH is much higher than the number of persons actually treated (Jaraj et al., 2014) and given this existing therapy, it is important to diagnose those patients who will respond well to ETV or shunt surgery (Stein et al., 2006). However, the diagnosis remains challenging since there are no distinctive pathognomonic features for idiopathic NPH and symptoms overlap with other dementias (Stein et al., 2006; Panagiotopoulos et al., 2005; Leinonen et al., 2012). It has even been suggested that the definitive diagnosis of this condition should be based on the patient's response to the surgery itself (Mori et al., 2012). At present, NPH is diagnosed by physical examination (gait evaluation) and computed tomography (CT) or magnetic resonance imaging (MRI) of the brain and the likelihood of positive response to therapy is determined using a lumbar puncture (Mori et al., 2012). Patients who show clinical improvement (as measured by improvement in gait) following removal of some CSF by lumbar puncture become candidates for shunt placement (Kang et al., 2014) or ETV. However, lumbar puncture has shown to have very low negative predictive value (<20%), as a number of patients improve after a shunt-placement despite a negative lumbar puncture test (Wikkelsø et al., 2012). Hence, a more sensitive diagnostic method is needed to identify more treatment-responsive NPH patients.

Given the constant production of CSF, it is critical that its circulation remains uninterrupted. The circulation of CSF can be obstructed at any point in the flow pathway, although the foramina of Monro (which connect each of the lateral ventricles to the third ventricle) and the cerebral aqueduct (connecting the third and fourth ventricles) are well-known anatomic bottlenecks because they are intrinsically predisposed to blockage (Nolte, 2009). An obstruction in either or both of the foramina of Monro causes the corresponding lateral ventricle to expand and become hydrocephalic, without distorting the remainder of the ventricular system. On the other hand, an obstruction in the cerebral aqueduct causes expansion of the third ventricle and both lateral ventricles (Nolte, 2009). Disproportionate expansion of components of the ventricular system could therefore give valuable clues about the specific point of CSF obstruction, which could in turn have an impact on whether ETV or shunt surgery will be effective (Yamada et al., 2015; Benedetto et al., 2017). This line of reasoning leads to the motivation behind our work since accurate automated segmentation and labeling tools for the multiple different compartments of the ventricular system of patients with severely enlarged ventricles are not presently available. The proposed method addresses this gap by providing a labeling method that enables more sophisticated evaluations of the ventricular system in neurodegenerative diseases, cerebrospinal fluid disorders, as well as in normal aging.

The major challenge when parcellating and labeling brains with severe ventriculomegaly is that the ventricles in these patients are both greatly enlarged and distorted from their normal anatomical shape, which makes accurate labeling of the major compartments challenging (see for example the lateral and third ventricles in Fig. 1(b), left image). Labeling the ventricular system as one component in healthy subjects using MRI is carried out routinely by several algorithms and their associated software packages. Although labeling the ventricular system in NPH patients seems like a simple task, many of these methods fail when applied to NPH brains (Shiee et al., 2011; Roy et al., 2015). Furthermore, a key limitation of these methods is their inability to parcellate the ventricular system into multiple compartments in order to quantify disproportionate dilation of the different ventricular cavities.

**Fig 2:**
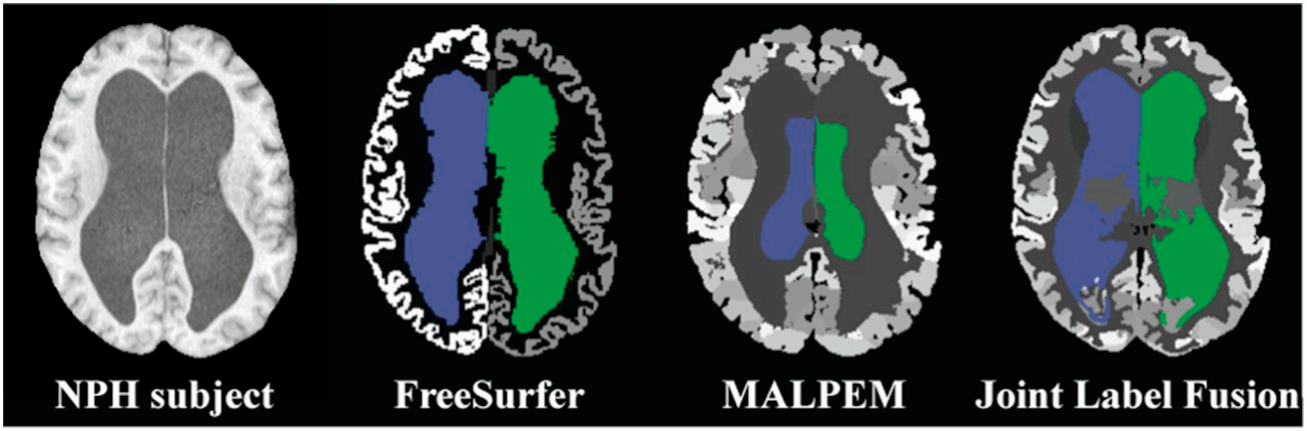
An example of a failed segmentation on a subject with severe ventriculomegaly, due to NPH.

Parcellation and labeling of the ventricular system into its four main compartments (i.e., left and right lateral and third and fourth ventricles) has been carried out by several approaches. Table 1 presents an overview of these segmentation algorithms. Multi-atlas label fusion methods have been shown to outperform other methods in the task of parcellating multiple brain structures, both on healthy and several (non-NPH) diseased populations (Babalola et al., 2009). However, these methods have a long runtime (several hours) and tend to fail in cases with enlarged ventricles, as demonstrated in Fig. 2. One reason for some of these failures is that most methods rely on registration between the atlas and subject images, which in pathological cases is rarely optimal. One recent algorithm, called RUDOLPH (Ellingsen et al., 2016; Carass et al., 2017; Shao et al., 2018a), was specifically designed to segment subjects with enlarged ventricles. Although robust in ventricle parcellation, this method takes hours to process a single image.

**Table 1:** Overview of brain segmentation methods.

Method	Whole ventricle label	ventricle parcellation	Remarks
volBrain (Manjón and Coupé, 2016)	✓		Non-local patch-based label fusion method. Provides online MRI brain volumetry system.
ALVIN (Kempton et al., 2011)	✓		Applies a binary mask to CSF segmented images using “unified segmentation” in SPM8 to segment the lateral ventricles.
TOADS (Bazinand and Pham, 2008)	✓		Segmentation framework based on both topological and statistical atlases of brain anatomy.
LoAD (Cardoso et al., 2011)	✓		Model-based segmentation method providing post refinements to a probabilistic segmentation model with anatomical tissue priors.
Adaptive Atlases (Shiee et al., 2011)	✓		Generates a subject specific atlas to segment brains with ventriculomegaly.
S3DL (Roy et al., 2015)	✓		Patch-based segmentation method using sparse dictionary learning.
FreeSurfer (Dale et al., 1999; Fischl et al., 2002; Fischl, 2012)	✓	✓	Atlas-based approach for whole brain segmentation.
MUSE (Doshi et al., 2016)	✓	✓	Multi-atlas label fusion method integrating optimal atlas selection strategy and a boundary modulation term to refine the segmentation.
BrainSuite (Shattuck and Leahy, 2002)	✓	✓	Atlas-based method for brain surface and volume labeling.
MALPEM (Ledig et al., 2015)	✓	✓	Multi-atlas label fusion method using a relaxation scheme to correct registration error.
NLSS (Asman and Landman, 2013)	✓	✓	Multi-atlas segmentation method with statistical fusion to incorporate intensity into the estimation process.
Joint label fusion (Wang et al., 2013)	✓	✓	Multi-atlas label fusion method formulating a weighted voting scheme to minimize the total expectation of the labeling error.
RUDOLPH (Ellingsen et al., 2016; Carass et al., 2017; Shao et al., 2018a)	✓	✓	Combines tissue segmentation and multi-atlas segmentation to correct registration priors. Designed for subjects with ventriculomegaly.

In recent years, deep learning—especially methods implemented with deep convolutional neural networks (CNNs)—has been used for MRI brain segmentation and has achieved state-of-the-art results (de Brebisson and Montana, 2015; Kayalibay et al., 2017; Chen et al., 2017; Dolz et al., 2017). For instance, (de Brebisson and Montana, 2015) used three orthogonal patches in a CNN to automatically segment 134 regions from brain MRIs. (Kayalibay et al., 2017) presented a 3D U-Net (Çiçek et al., 2016) to segment tumor regions in brain MRIs. (Chen et al., 2017) proposed a voxelwise residual network to segment gray matter, white matter, and CSF tissues from MRIs. (Dolz et al., 2017) investigated a 3D CNN with small kernels and deeper architectures to segment subcortical structure in brain MRIs. So far, skip connections have proven to be beneficial in biomedical image segmentation (Ronneberger et al., 2015; Drozdzal et al., 2016; Chen et al., 2017). The Long skip connections (Long et al., 2015; Ronneberger et al., 2015; Milletari et al., 2016; Çiçek et al., 2016) combine the high resolution features from the contracting path with the upsampled features from the expanding path to help recover the full spatial resolution at the output. The Short skip connections (He et al., 2016a, He et al., 2016b; Szegedy et al., 2017) create shortcuts between layers within a residual block to address the degradation problem in deeper networks.

In this paper, we investigated a 3D CNN with the U-Net architecture and residual blocks for brain ventricle parcellation using MRI. We used instance normalization (Ulyanov et al., 2017) to make the network invariant to linear transformation of image intensities. Furthermore, the network combined the feature maps created at different resolution levels to refine the final classification. A preliminary version of this work was reported in conference form (Shao et al., 2018b); here we present an improvement of this work with more extensive validation and comparisons.

We conducted comprehensive experiments over two data sets: one publicly available data set consisting of healthy controls and one data set of NPH patients. We trained and evaluated the network on MRIs from both data sets. Our method achieved competitive performance on the healthy controls. When evaluated on the NPH data, our method produced segmentation results that are significantly better than the state-of-the-art brain segmentation approaches in three evaluation metrics. Furthermore, existing methods usually take hours to run on each image whereas our approach runs in seconds per image.

## 2 Materials and methods

### 2.1 Data sets

Images from two separate data sets of T1-weighted (T1-w) magnetically prepared rapidly acquired gradient echo (MPRAGE) MRI scans were used to train and evaluate our method. The first data set comprised 50 MRIs of healthy controls (age range: 18–96 years with mean age of 44.54 years and standard deviation of 25.16 years) from the Open Access Series on Imaging Studies (OASIS) data set (Marcus et al., 2007). They were acquired on a 1.5 T Siemens scanner with scanner parameters of: TR = 9.7 ms, TE = 4.0 ms, FA = 10°, TI = 20 ms, and 1 × 1 × 1.25 mm^3^ voxel size. Written informed consent was obtained from all participants. The images were manually labeled by experts from Neuromorphometrics Inc. (NMM).^1^ The labeled images comprise 134 cortical and subcortical structures, including the left and right lateral ventricles, and the third and fourth ventricles. Since our algorithm focuses on ventricle parcellation, we converted the manual labels into five: the four ventricle labels and a background label representing the remaining parts of the image.

The second data set contained 95 T1-w MPRAGE MRIs from our own NPH database (age range: 26–90 years with mean age of 66.83 years and standard deviation of 15.08 years). They were acquired on a 3 T Siemens scanner with typical parameters (with small variations): TR = 2110 ms, TE = 3.24 ms, FA = 8°, TI = 1100 ms, and 0.859 × 0.859 × 0.900 mm^3^ voxel size. The acquired data was retrospectively included in our study with local institutional review board approval. The four ventricles of the 95 NPH images were manually delineated. This was done by first identifying the anatomical structure of the ventricles, which required 3–4 h per patient. These were reviewed by separate experts in neuroanatomy, with either minor editing or returned to the delineator for correction. Once a ventricular system mask was agreed, the components of right and left lateral ventricles, both foramina of Monro, third ventricle, cerebral aqueduct, and fourth ventricle were identified. This manual parcellation of the ventricular system took another hour per patient to complete. The cerebral aqueduct and the foramina of Monro are not included within our testing, as there do not currently exist such detailed anatomical atlases of the ventricular system in use elsewhere. Thus for evaluation purposes, the foramina of Monro is included with the corresponding lateral ventricle, and the cerebral aqueduct with the fourth ventricle, making the labeling comparable with NMM.

### 2.2 Ventricle parcellation network (VParNet)

**Fig. 3:**
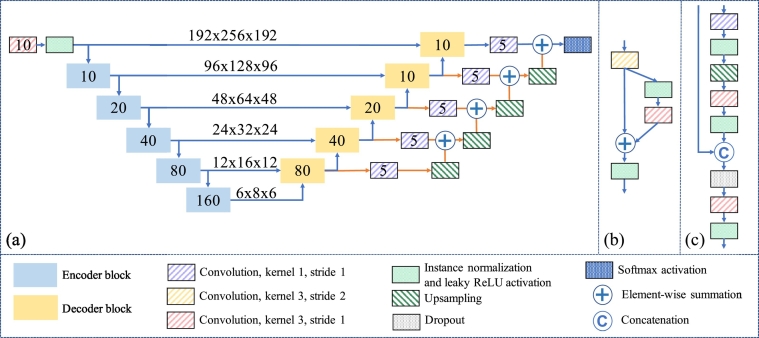
(a) Architecture of the proposed ventricle parcellation network (VParNet). The numbers in the encoder and decoder blocks indicate the number of output channels. The shape of the tensor is denoted at each resolution level. (b) Architecture of the encoder block. (c) Architecture of the decoder block.

Given an MRI of a human brain, our goal is to assign a particular label to each voxel. In this current work, we use a total of five labels to parcellate the T1-w image, and these labels consist of four ventricle labels and a background label. Our network, referred to as the Ventricle Parcellation Network, or VParNet, was modified from (Kayalibay et al., 2017) and designed to label each voxel with one of the five labels. It consists of a contracting path with a series of encoder blocks and an expanding path with a series of decoder blocks, as illustrated in Fig. 3. The long skip connections from the encoder blocks provide the high-resolution features to their matching decoder blocks through concatenation (Çiçek et al., 2016). The 1 × 1 × 1 projection convolutions connected to each decoder block reduce the number of output channels to the number of labels, which is five in our case. The details of the different blocks are described below.

#### 2.2.1 Encoder block

The encoder block in the contracting path, as shown in Fig. 3(b), is similar to the residual unit in (He et al., 2016b). We refer to the instance normalization layer (Ulyanov et al., 2017) and leaky rectified linear unit (ReLU) layer (Maas et al., 2013) as activation layers. The input feature map of each encoder block goes through a 3 × 3 × 3 convolution layer with stride setting to two, activation layers, a 3 × 3 × 3 convolution layer, and activation layers. The skip connections within the blocks act as identity maps. Their outputs are combined with the outputs of the second convolution layer using element-wise summation. The short skip connections make error reduction faster and increase accuracy (He et al., 2016a). Compared to batch normalization, instance normalization can make the feature maps invariant to linear transformation of the input image intensities (Ulyanov et al., 2017).

#### 2.2.2 Decoder block

The decoder block in the expanding path, as shown in Fig. 3(c), consists of a 1 × 1 × 1 convolution layer, activation layers, an upsampling layer, a 3 × 3 × 3 convolution layer, activation layers, a concatenation layer, a dropout layer, a 3 × 3 × 3 convolution layer, and activation layers. In the upsampling layer, the coarse feature map is upsampled to a finer resolution by repeating the data in three dimensions. The upsampling layer is followed by a convolution and activation layers, whose output is concatenated with the output feature map from the matching encoder block within the concatenation layer. The dropout layer is used to regularize the network (Srivastava et al., 2014).

#### 2.2.3 Classification block

The final decoder block is followed by the activation layers and a 1 × 1 × 1 convolution layer to reduce the number of channels to 5, i.e., the number of output labels. The classification result is refined by combining feature maps created from multiple resolution levels (Szegedy et al., 2015; Kayalibay et al., 2017; Dolz et al., 2017). This step encourages the features extracted at different layers to be consistent. The multi-level feature maps are combined in the following way: the feature maps from different levels go through a 1 × 1 × 1 convolution layer to become 5-channel feature maps. Then the 5-channel feature map from the lowest resolution level is upsampled to have the same resolution as the second-lowest feature map. These two are combined via element-wise summation. The combined feature map is upsampled to a higher resolution level and added to the feature map at this resolution level. The upsampling and summation are done sequentially until we get a feature map with the highest resolution, as shown by the orange arrows in Fig. 3(a). The last summation operation is followed by a softmax activation layer, converting the feature map to a probability map for the final classification.

### 2.3 Data pre-processing and implementation

All the images from the NMM and NPH data sets were pre-processed through N4 inhomogeneity correction (Tustison et al., 2010), rigid registration to MNI 152 atlas space (Fonov et al., 2009), and skull-stripping (Roy et al., 2017). The original image size after pre-processing was 241 × 286 × 241 voxels; however, each image was symmetrically cropped around the brain mask to 192 × 256 × 192 voxels to reduce the number of background voxels before being input into VParNet.

The VParNet was trained on images from both the NMM and NPH data sets. Of the 50 MRIs from the NMM data set, 15 were used for training, 5 were used for validation, and the remaining 30 were used for testing. Of the 95 NPH images, 25 were chosen for training, 5 for validation, and the remaining 65 were used for testing. The 95 NPH images were sorted by the volume of the ventricular system and the 25 training MRIs were chosen such that they covered the entire spectrum of ventricle sizes in the NPH cohort. Of all the images in our two data sets, 28% were used for training, 7% were used for validation, and the remaining 65% were used for testing.

**Fig. 4:**
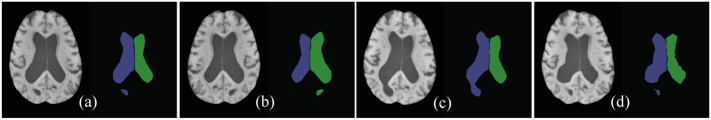
Data augmentation examples (MRI and the corresponding label image). (a) The original image; (b) Left-right flipping; (c) Random rotation; (d) Elastic deformation.

Due to the limited amount of available training data, we augmented the training data by introducing left-right flipping, random rotation, and elastic deformation. Examples of data augmentation are shown in Fig. 4. The leaky ReLU negative slope was 0.1 and the dropout rate was 0.2. The loss function used to train the network was one minus the mean Dice similarity coefficient (DSC) (Dice, 1945) of all the labels, which is defined as

$$\text{Loss}=1-\frac{1}{L}\sum_{l=1}^{L}\frac{\epsilon +2\sum_{i}P_{\mathrm{il}}T_{\mathrm{il}}}{\epsilon +\sum_{i}P_{\mathrm{il}}+\sum_{i}T_{\mathrm{il}}},$$

where *l* ∈ {1,  … , *L*} is the label index, *L* is the total number of labels, *P*_*il*_ is the probability that voxel *i* belongs to the label *l* generated by the network, and *T*_*il*_ is the binary value indicating if voxel *i* belongs to the label *l* based on the manual delineations. The ϵ is used to avoid zero denominator, and it was set to 10^−3^ during training. Deep networks use a gradient descent algorithm to update the network parameters. Gradient descent is an iterative optimization algorithm for finding the minimum of an objective function. In our application, we want to maximize DSC, which measures the similarity between the network prediction and the manual delineation. Therefore, setting “1-DSC” as our loss function will minimize “1-DSC” during training, which simultaneously maximizes DSC.

The VParNet was initially trained for 200 epochs using the Adam optimizer (Kingma and Ba, 2014) with step size *α* = 0.001, the exponential decay rates *β*_1_ = 0.9, *β*_2_ = 0.999, and *ε* = 10^−7^. During each epoch, the original 40 images (15 from NMM and 25 from NPH) were first left-right flipped to create another set of 40 images. Then the 80 images were randomly rotated along each axis and deformed to create an additional 160 images. In each epoch, these 240 (80 + 160) images were used to optimize the network parameters, with a batch size of one. We observed that the performance of the trained network on the validation data did not improve after 150 epochs. Therefore, we evaluated the network after 150 epochs.

### 2.4 Experimental setup

The performance of the VParNet was compared with four state-of-the-art brain segmentation methods: FreeSurfer (version 6.0.0) (Dale et al., 1999; Fischl et al., 2002; Fischl, 2012), MALPEM (Ledig et al., 2015), Joint label fusion (JLF) from the ANTs software package (Wang et al., 2013; Wang and Yushkevich, 2013), and RUDOLPH (Ellingsen et al., 2016; Carass et al., 2017; Shao et al., 2018a). We also conducted an ablation analysis of the VParNet to see how different strategies affect the performance. The details of these network variations are reported in Table 2.

**Table 2:** Ablation analysis overview.

	Encoder	Normalization	Data augmentation	Combine multi-level feature maps
CNN-1	Residual	Instance		✓
CNN-2	Residual	Batch	✓	✓
CNN-3	Plain	Instance	✓	✓
CNN-4	Residual	Instance	✓	
VParNet	Residual	Instance	✓	✓

### 2.5 Evaluation metrics

In our experiments, we used the Dice similarity coefficient (DSC) (Dice, 1945), 95% Hausdorff distance (HD) (Dubuisson and Jain, 1994), and absolute volume difference (AVD) to evaluate the accuracy of the ventricle parcellation results.

#### 2.5.1 Dice similarity coefficient

The Dice similarity coefficient (DSC) is a volume-based metric broadly used to evaluate segmentation results. The DSC between two binary segmentation masks *S* and *T* is defined as

$$\mathrm{DSC}=\frac{2|S\cap T|}{|S|+|T|}.$$

The DSC values are in the range [0, 1], where 1 indicates perfect overlap between *S* and *T* and 0 indicates no overlap at all. We calculated the DSC between the automatic segmentations and the manual delineations to evaluate the performances of the different methods.

#### 2.5.2 95% Hausdorff distance

Hausdorff distance (HD) is a distance-based metric used to measure the boundary distance between two segmentations. It is the longest of all the distances from a point in one point set to the closest point in the other point set. The HD between two sets of points *A* and *B* can be defined as

$$\mathrm{HD}=\max\left\{\max_{a\in A}\;d\left(a,B\right),\max_{b\in B}\;d\left(b,A\right)\right\},$$

where *a* and *b* are points from sets *A* and *B*, respectively, and *d*(*a*, *B*) is the distance between a point *a* and the set *B*, which is defined as:

$$d\left(a,B\right)=\min_{b\in B}∥a-b∥.$$

Lower values of HD indicate higher segmentation accuracy. We used the 95% distance to calculate the HD, since HD is sensitive to outliers.

#### 2.5.3 Absolute volume difference

The absolute volume difference (AVD) between the volume of the segmentation result *V*_*S*_ and the volume of the ground truth *V*_*T*_ is defined as

$$\mathrm{AVD}=\frac{|V_{S}-V_{T}|}{V_{T}}\times 100\%.$$

Lower values of AVD indicate better volumetric agreement.

## 3 Results

The objective of the proposed method is to provide accurate parcellation of the ventricular system in both healthy controls and patients with mild to severe cases of ventriculomegaly. All the methods (FreeSurfer, MALPEM, JLF, RUDOLPH, and VParNet) were run in an automatic manner.

### 3.1 Ventricle parcellation of healthy brains - quantitative evaluation using manual ventricle labels

In this experiment, we ran each method on the 30 testing subjects from the NMM data set, investigating their performances on healthy subjects. Although MALPEM and FreeSurfer have built-in skull-stripping methods, we provided our skull-stripped data to them for consistency with the processing; we note that in our experience, FreeSurfer produced better results with our provided skull-stripped data than the built-in skull-stripping. The 15 NMM images used to train the networks served as the atlases for MALPEM, JLF, and RUDOLPH. We used symmetric image normalization (SyN) (Avants et al., 2008) for non-rigid registration in JLF and RUDOLPH.

**Fig. 5:**
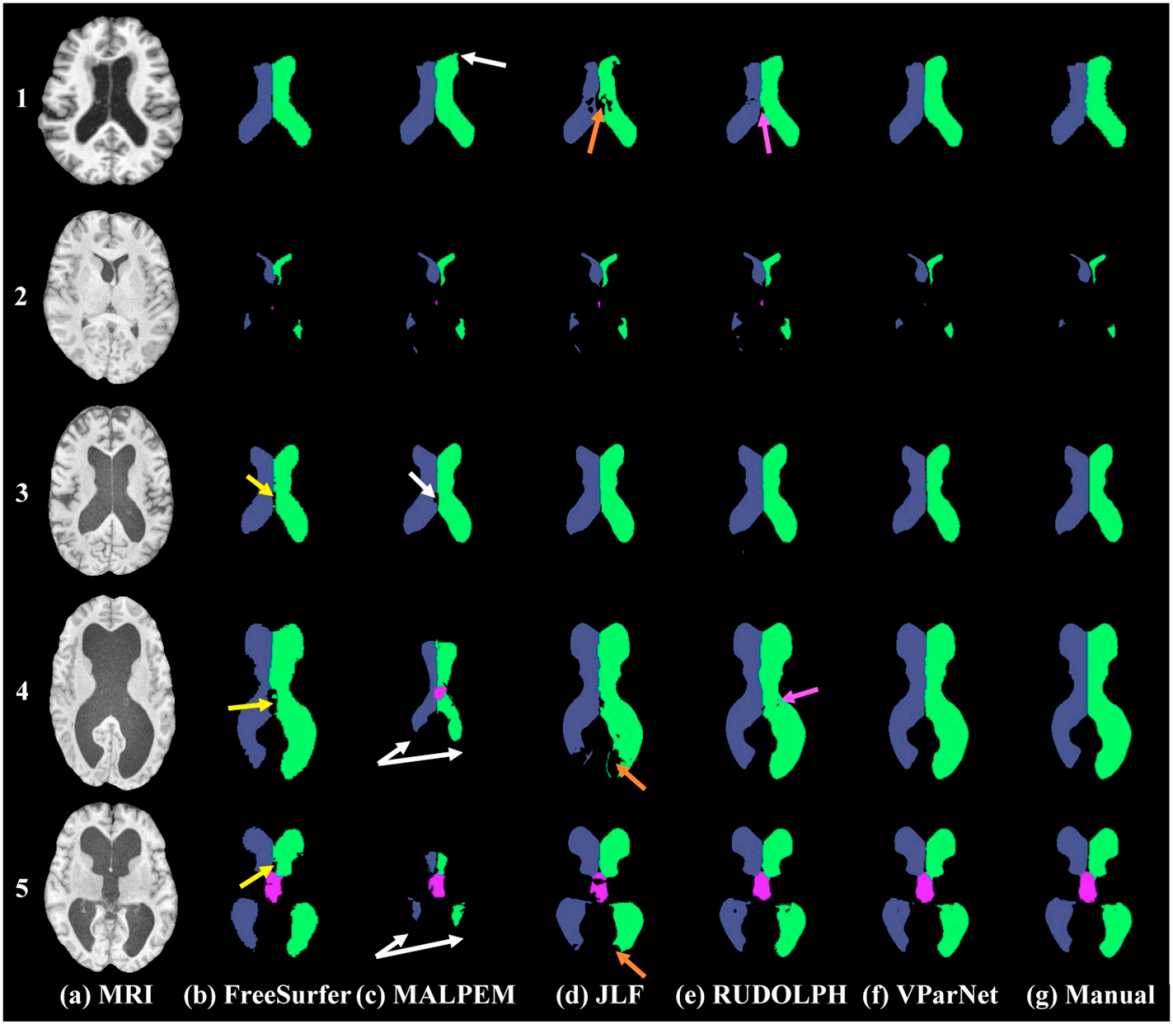
Visual comparison of the five segmentation methods for one NMM (Row-1) and four NPH subjects (Row-2 through Row-5): (a) T1-w MPRAGE image; (b) FreeSurfer; (c) MALPEM; (d) Joint Label Fusion (JLF); (e) RUDOLPH; (f) VParNet; and (g) manual rater. In Row-1, white arrow: over-segmentation of the left lateral ventricle from MALPEM; orange and magenta arrows: failed segmentation on the ventricle boundaries near the septum pellucidum from JLF and RUDOLPH, respectively. In Row-3 through Row-5, the yellow, white, and orange arrows: inaccurate boundaries segmentation from FreeSurfer, MALPEM, and JLF, respectively. In Row-4, the magenta arrow: slightly under-segmentation of the ventricle from RUDOLPH.

**Fig. 6:**
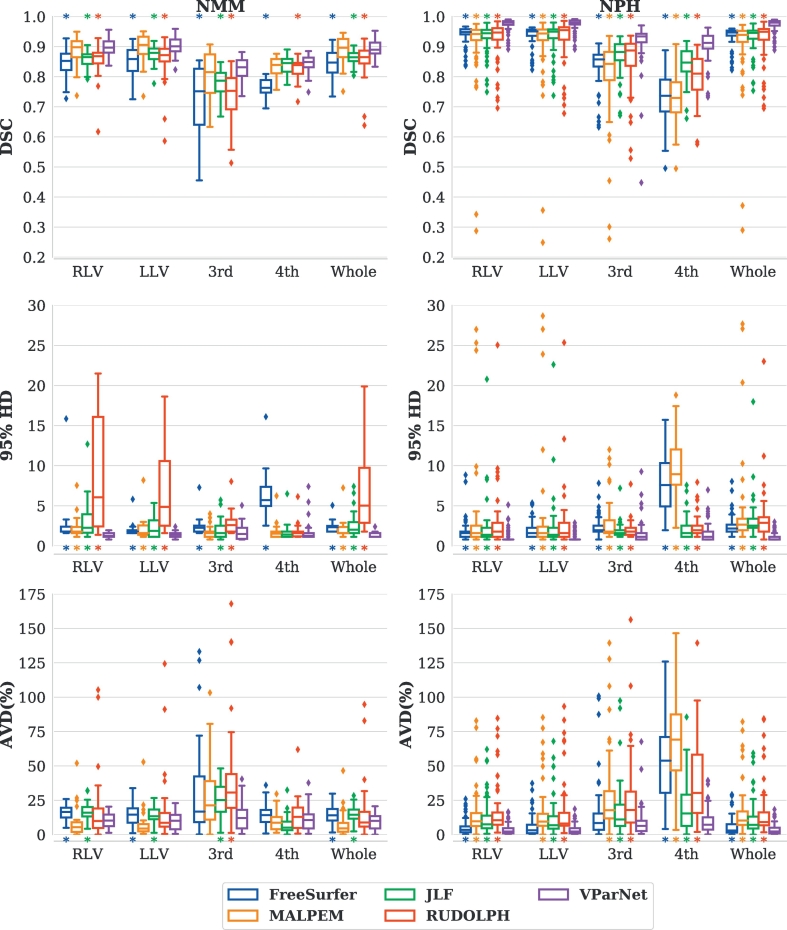
Boxplots of the Dice similarity coefficient (DSC), 95% Hausdorff distance (HD), and absolute volume difference (AVD) over 30 T1-w MRIs from the NMM data set (left side) and 65 T1-w MRIs from the NPH data set (right side). Ventricular system key: Right lateral ventricle (RLV), left lateral ventricle (LLV), third ventricle (3rd), fourth ventricle (4th), and whole ventricular system (Whole). A paired Wilcoxon signed-rank test with an *α*-level of 0.005 was conducted to compare VParNet with each of the other methods. The asterisk at the top/bottom of each box means the corresponding evaluation metric of FreeSurfer/MALPEM/JLF/RUDOLPH is significantly different (*p* ‐ value < 0.005) from VParNet results.

A visual comparison of the ventricle parcellation results produced by the five methods is shown in Row-1 of Fig. 5. We only present the ventricle labels of the results from FreeSurfer, MALPEM, JLF, and RUDOLPH. We observe that MALPEM slightly over-segmented the left lateral ventricle (see the white arrow in Row-1 of Fig. 5), and JLF and RUDOLPH did not capture the ventricle boundaries near the septum pellucidum on this subject (see the orange and magenta arrows in Row-1 of Fig. 5). Boxplots of the DSC, 95% HD, and AVD over 30 testing MRIs from the NMM data set are shown in Fig. 6, left side. We conducted a paired Wilcoxon signed-rank test (Wilcoxon, 1945) with an *α*-level of 0.005 and mark the significant difference between VParNet and each of the other methods in asterisks at the top/bottom of each plot. VParNet outperformed FreeSurfer in each evaluation metric, except AVD of the third and fourth ventricles. Comparing to MALPEM, VParNet achieved competitive results in terms of DSC and AVD, and better results on the lateral ventricles in terms of 95% HD. Comparing to JLF and RUDOLPH, VParNet produced overall higher DSC and lower 95% HD and AVD.

### 3.2 Ventricle parcellation of NPH patients - quantitative evaluation using manual ventricle labels

In this experiment, we ran each method on the 65 testing subjects from the NPH data set. In FreeSurfer, we turned the “bigventricles” switch “on” to account for the enlarged ventricles. For the non-rigid registration method SyN in JLF and RUDOLPH, we set the step size of the gradient descent to 0.3 to enable larger deformations.

Examples of the ventricle parcellation results obtained from the five methods on NPH subjects are shown in Fig. 5, Row-2 to Row-5. The subject in Row-2 is a mild NPH case with relatively small ventricles. MALPEM, JLF, and RUDOLPH tended to over-segment the ventricles of this subject, while VParNet performed better on the boundaries. VParNet produced smoother segmentations than FreeSurfer. Row-3 shows a moderate case of NPH, where most methods performed well on ventricle parcellation. The yellow and white arrows point to inaccurate segmentations on the boundaries from FreeSurfer and MALPEM, respectively. Rows-4 and 5 show NPH subjects with severely enlarged ventricles. FreeSurfer, MALPEM, and JLF failed to identify the boundaries of the enlarged ventricles (see the yellow, white, and orange arrows), and RUDOLPH slightly underestimated the size of the ventricles (see the magenta arrow).

Boxplots of DSC, 95% HD, and AVD are presented in Fig. 6, right side. The proposed VParNet performed better than all the other methods on each ventricle label in terms of DSC and 95% HD. These results were statistically significant in a paired Wilcoxon signed-rank test with an *α*-level of 0.005. Regarding AVD, VParNet significantly outperformed the other methods on each label, except on the left lateral ventricle and the third ventricle results generated by FreeSurfer, where the difference did not reach statistical significance. In summary, these boxplots demonstrate that VParNet produced more accurate ventricle parcellation results on subjects with highly variable ventricle sizes, from healthy to severe ventriculomegaly.

### 3.3 Evaluation of network variations

We conducted an ablation analysis of the VParNet to see how different strategies affect the performance of the network (see Table 2 for the details of the network variations). Table 3 shows the results of the ablation analysis. The training set was the same in each network, except that the training images were white matter peak normalized before use in CNN-2, which used batch normalization instead of instance normalization.

**Table 3:** The mean DSC, 95% HD, and AVD (standard deviation) over 95 testing images (30 from NMM and 65 from NPH). Ventricular system key: Right lateral ventricle (RLV), left lateral ventricle (LLV), third ventricle (3rd), fourth ventricle (4th), and whole ventricular system (Whole). A paired Wilcoxon signed-rank test with an *α*-level of 0.005 was conducted to compare VParNet with each of the other networks. The asterisk means the corresponding evaluation metric of CNN-1/CNN-2/CNN-3/CNN-4 is significantly different (*p* ‐ value < 0.005) from VParNet results.

	RLV	LLV	3rd	4th	Whole
DSC:					
CNN-1	0.944^∗^(0.05)	0.947^∗^(0.04)	0.880^∗^(0.06)	0.875^∗^(0.05)	0.944^∗^(0.04)
CNN-2	0.946^∗^(0.05)	0.947^∗^(0.05)	0.887(0.07)	0.875(0.06)	0.944^∗^(0.05)
CNN-3	0.948^∗^(0.04)	0.951(0.04)	0.891(0.07)	0.881(0.05)	0.947(0.04)
CNN-4	0.949(0.04)	0.951(0.04)	0.887(0.07)	0.882(0.06)	0.948(0.04)
VParNet	0.950(0.04)	0.951(0.04)	0.887(0.08)	0.884(0.05)	0.948(0.04)

95% HD:					
CNN-1	1.738^∗^(1.7)	1.573^∗^(1.1)	1.727^∗^(1.0)	1.958(1.5)	1.454^∗^(1.2)
CNN-2	1.367^∗^(0.99)	1.281(0.89)	1.641(1.1)	1.663(1.3)	1.311^∗^(0.80)
CNN-3	1.232(0.79)	1.158(0.53)	1.489(1.0)	2.730(11)	1.238(0.62)
CNN-4	1.393(2.2)	1.143(0.48)	1.990^∗^(2.4)	1.689(1.3)	1.162(0.49)
VParNet	1.173(0.65)	1.143(0.48)	1.620(1.3)	1.648(1.2)	1.174(0.50)

AVD (%):					
CNN-1	5.54(5.2)	5.26(5.6)	12.3^∗^(11)	11.0(9.8)	5.37(5.1)
CNN-2	6.51(6.7)	6.68^∗^(6.8)	10.1(10)	13.1(11)	6.66^∗^(6.6)
CNN-3	6.02^∗^(5.6)	5.80^∗^(5.6)	9.85(9.1)	11.5(9.0)	5.90^∗^(5.5)
CNN-4	5.76(5.1)	5.45^∗^(5.4)	10.5(9.9)	11.1(9.1)	5.57(5.1)
VParNet	5.62(5.2)	5.54(5.5)	10.4(11)	10.4(9.0)	5.55(5.2)

When comparing CNN-1 and VParNet, we observe that adding data augmentation improves the parcellation performance in terms of both DSC and 95% HD. The improvements show significance in a paired Wilcoxon signed-rank test with an *α*-level of 0.005. The comparison between CNN-2 and VParNet demonstrates that using instance normalization produces comparable parcellation results with the network using batch normalization. The performance of the instance normalization comes from the training and testing not requiring intensity normalization. Comparing to CNN-3, VParNet has broadly higher DSC and lower 95% HD and AVD. The results show significant improvements on the AVD of the lateral ventricles, indicating the contribution of the short skip connection within the encoder block. Comparing CNN-4 and VParNet, both networks converge to similar validation loss. However, VParNet used only 5 epochs to reduce the validation loss to 0.1, while CNN-4 used 24 epochs. This shows that combining multi-level feature maps does effectively speed up network convergence.

## 4 Discussion

We have performed a comprehensive evaluation of our proposed VParNet using data from two different data sets, i.e. NMM and NPH. The accuracy of the ventricle parcellation was evaluated using DSC, 95% HD, and AVD between the automated parcellation and manual delineations. VParNet was trained on 15 healthy controls and 25 subjects with NPH. When testing on the NMM data set, our method achieved competitive parcellation results compared to state-of-the-art brain segmentation algorithms, as shown in Fig. 6, left side. The NPH data set was then used to demonstrate the robustness of VParNet to subjects with highly variable ventricle sizes and shapes. Our method produced significantly better results than the other methods in terms of DSC, 95% HD and AVD, as shown in Fig. 6, right side. As shown in the boxplots, our method achieved better evaluation scores and lower standard deviations, demonstrating the consistency of the network performance on healthy and NPH subjects.

Considering the performance of VParNet on the two data sets, the network obtained overall higher DSC and lower AVD in the NPH data set compared to the healthy control data set. The reason is that these two metrics are biased by the size of the segmented structure. In the NMM data set, the ventricular volume is smaller, ranging from 12 ml to 132 ml with mean value being 51 ml, while the ventricular volume in the NPH data set ranges from 10 ml to 400 ml with mean value being 120 ml. A similar bias is seen when considering the size of different ventricular compartments; VParNet reached a better evaluation score on the lateral ventricles than the third and the fourth ventricles in terms of DSC and AVD.

When exploring the variations of the network, the accuracy of VParNet results was statistically higher than the network without data augmentation. This demonstrates the value of data augmentation in tasks with limited amounts of training data. VParNet and the network without combining multi-level feature maps converged to a similar validation loss. However, VParNet converged much faster, indicating the importance of incorporating features at different levels. We also trained a network with batch normalization using the training data without white matter peak normalization; however, this network showed poor generalization, demonstrating the strength of instance normalization that does not require intensity normalization of the input images.

Automated parcellation of the ventricular system in MRIs of patients with enlarged ventricles is challenging due to the variations of ventricle volumes and shapes across individual subjects. Many approaches have been proposed to segment the human brain in MRI, and some of them are capable of parcellating the ventricular system into its four main cavities (see Table 1). Conventional atlas-based methods require deformable image registration, which is typically time-consuming. Moreover, these methods rely on good registration results between the atlas and subject images, however, the enlarged ventricles can cause significant registration errors. FreeSurfer has a special “bigventricles” option to account for enlarged ventricles in hydrocephalus and late stage Alzheimer's cases. It is worth noting that we used this special option when running FreeSurfer on the NPH data set. We also experimented with running FreeSurfer in its default setting and on some subjects with severely enlarged ventricles, the program failed to output segmentation results at all. For this FreeSurfer experiment, the mean DSC of the whole ventricular system on the remaining testing images was 0.77, while in FreeSurfer with “bigventricles” flag, the mean DSC was 0.94.

In our experiments, we also processed our data with BrainSuite (Shattuck and Leahy, 2002), MUSE (Doshi et al., 2016), and NLSS (Asman and Landman, 2013). BrainSuite produced segmentation results with mean DSC of 0.6 in the NPH data set, which did not compare favorably with the other methods. MUSE can be run remotely on a web platform; however, we were not permitted to upload our NPH data due to health care privacy considerations. The multi-atlas based method NLSS takes about 36 h to process one subject, which was not efficient and made it challenging for us to complete the entire test data set. Therefore, we did not report comparisons of these methods to the proposed network here.

A few studies that use deep learning approaches to parcellate the ventricular system (or parts thereof) have been reported in the literature, including (Ghafoorian et al., 2018; Huo et al., 2019); and (Atlason et al., 2019). (Ghafoorian et al., 2018) utilized a 2D fully convolutional network based on the U-Net architecture to segment the left and right lateral ventricles using T1-w and FLAIR images. They reported a mean DSC of 0.874 for both left and right lateral ventricles on a longitudinal data set. (Ghafoorian et al., 2018) did not parcellate any other portion of the ventricular system, as we do. Their reported DSC for the lateral ventricles—though on a different cohort—is below the DSC we achieved for the same structure. (Huo et al., 2019) developed a spatially localized atlas network to do whole brain segmentation. The network was trained on 45 T1-w MRIs with manual labels and 5111 multi-site T1-w MRIs labeled by a multi-atlas segmentation method. They reported a mean DSC of 0.821, 0.831, 0.814, and 0.475 on right and left lateral, 3rd, and 4th ventricles, respectively. Both (Ghafoorian et al., 2018) and (Huo et al., 2019) were not specially designed for and have not been tested on patients with enlarged ventricles. (Atlason et al., 2019) developed a patch-based 3D U-Net CNN using T1-w and T2-w MRIs for labeling the brain ventricular system in patients with ventriculomegaly. The training labels were generated by an automated whole brain segmentation algorithm. They reported preliminary results on a small data set with mean DSC of 0.906 on the entire ventricular system. Our reported mean DSC for the same structure is 0.948, again with the caveat that the cohorts are different.

The typical processing time on CPU-base implementation of the five methods is reported in Table 4. The programs were run on a 16-core 2.4 GHz CPU. A CPU version of VParNet required about two minutes to process one image, which is much lower than the several hours required by the other methods. We note that this processing time can be brought down to approximately 30 s if running on an NVIDIA Tesla K80 GPU.

**Table 4:** Processing time comparison of different methods.

Method	Runtime per image
FreeSurfer	9.5 h
MALPEM	2 h
JLF	15 h
RUDOLPH	15 h
VParNet	2 min

The software will be made publicly available, so that other researchers can evaluate the method on different patient populations. We have shown results on data from two different sites using scans from different MRI manufacturers so we believe that with further training it can be applied in multi-site studies using training data from each site. The proposed network is also robust to white matter hyperintensities (WMH), which are often associated with NPH and located adjacent to the lateral ventricles. WMH can sometimes negatively affect the outcome of automatic segmentation algorithms (Atlason et al., 2019). In our NPH testing data, 59 of 65 subjects have WMH close to the lateral ventricles and by visually examining the results, we found the proposed method provided accurate parcellation of the ventricular system in the presence of WMH.

Evaluations of the ventricular system plays a significant role for diagnosing neuro-degenerative diseases and cerebrospinal fluid disorders. Our method provides a tool for automated parcellation of the ventricular system in healthy subjects and patients with ventriculomegaly, enabling more sophisticated analyses of the ventricular structures in both health and disease.

## 5 Conclusions

In this work, we presented a 3D U-Net for ventricle parcellation of brains with enlarged ventricles from MRI. We incorporated the deep residual learning technique in the encoder block to ease the degradation problems in training. The refinement of the classification that combines feature maps from multiple resolution levels can speed up network convergence. We used instance normalization instead of batch normalization to make the network invariant to linear transformation of the image intensities. Thus the training and testing images can be sent to the network without intensity normalization.

We presented results on both healthy controls and NPH patients. To the best of our knowledge, this is the first deep learning method specifically developed to parcellate the ventricular system of the human brain. In particular, we have demonstrated its utility on the full specturm of ventricular system sizes—from healthy controls and mild enlargement due to normal aging to dramatically enlarged because of hydrocephalus, see Fig. 5 for examples. We showed that our proposed network achieves state-of-the-art performance on a healthy data set and significantly better performance than top competing methods on an NPH data set. The evaluation indicated the robustness of our network to high variability in ventricle shapes and sizes; from healthy to severe ventriculomegaly.

## Appendix A Supplementary data

Supplementary material. Image 1

## Footnotes

1 http://www.neuromorphometrics.com/
