# Supplementary material for: Brain ventricle parcellation using a deep neural network: Application to patients with ventriculomegaly
Source: Neuroimage Clin. 2019 May 24;23:101871. doi: 10.1016/j.nicl.2019.101871 (PMC6551563; doi:10.1016/j.nicl.2019.101871)
Supplement: Supplementary file 1 — Supplementary material. [file mmc1.pdf]

Brain ventricle parcellation using a deep neural network: Application to patients with ventriculomegaly: Supplementary materials

Muhan Shao[a,*]

[a]Department of Electrical and Computer Engineering, The Johns Hopkins University, Baltimore, MD 21218, USA

1. Detailed quantitative results of ventricle parcellation

The quantitative evaluations of FreeSurfer (Dale et al., 1999; Fischl et al., 2002; Fischl, 2012), MALPEM (Ledig et al., 2015), Joint Label Fusion (JLF) (Wang et al., 2013), RUDOLPH (Ellingsen et al., 2016; Carass et al., 2017; Shao et al., 2018a), and the proposed VParNet over two data sets (NMM and NPH) are presented in Table 1. The evaluation metrics are Dice similarity coefficient (DSC), 95% Hausdorff distance (HD), and absolute volume difference (AVD). We conducted a paired Wilcoxon signed-rank test (Wilcoxon, 1945) with an α-level of 0.005 and mark the significant difference between VParNet and each of the other methods in asterisks.

2. Five-fold cross validation

We applied 5-fold cross validation on our data sets to evaluate the proposed network. The 50 MRIs from the NMM data set and the 95 MRIs from the NPH data set were split into 5 groups. Each group contained 10 NMM images and 19 NPH images. Firstly, we sorted the 50 NMM images and 95 NPH images separately by the volume of the ventricular system. Secondly, the sorted NMM and NPH data sets were evenly divided into 10 and 19 small groups, respectively. Each small group contained 5 MRIs with similar ventricle sizes. Thirdly, for each small group, we randomly permutated the 5 MRIs and assigned them to our final 5 groups (one image per group). Therefore, each of the 5 cross-validation groups will cover a wide spectrum of ventricle sizes from both data sets.

During cross validation, we trained the VParNet 5 times using different training data. The network trained in the ith time, which we referred to as TestFold-i, took the

*See the main text for the complete author list.
Please address correspondence to:
Muhan Shao,
Department of Electrical and Computer Engineering, The Johns Hopkins University, 105 Barton Hall, 3400 N. Charles St., Baltimore, MD 21218, USA
Email address: muhan@jhu.edu (Muhan Shao)

Table 1: The mean (standard deviation) of Dice similarity coefficient (DSC), 95% Hausdorff distance (HD), and absolute volume difference (AVD) over 30 T1-w MRIs from the NMM data set and 65 T1-w MRIs from the NPH data set. The asterisks mean the results of the corresponding method (FreeSurfer, MALPEM, JLF, and RUDOLPH) are significantly different (p-value < 0.005) from VParNet results.

	**RLV**	**LLV**	**3rd**	**4th**	**Whole**
**NMM:**					
**DSC:**					
**FreeSurfer**	0.847*(0.05)	0.852*(0.04)	0.729*(0.22)	0.763*(0.03)	0.845*(0.05)
**MALPEM**	0.889(0.05)	0.897(0.04)	0.803(0.08)	0.833(0.03)	0.891(0.04)
**JLF**	0.859*(0.03)	0.877*(0.03)	0.779*(0.04)	0.837(0.03)	0.865*(0.02)
**RUDOLPH**	0.851*(0.07)	0.857*(0.07)	0.735*(0.09)	0.827*(0.04)	0.853*(0.06)
**VParNet**	0.898(0.03)	0.900(0.03)	0.824(0.04)	0.840(0.03)	0.895(0.03)
**95% HD:**					
**FreeSurfer**	2.496*(2.6)	1.991*(0.77)	2.432*(1.0)	6.204*(2.6)	2.404*(0.68)
**MALPEM**	2.157*(1.3)	2.063*(1.3)	1.708(0.72)	1.728(1.0)	2.051*(1.1)
**JLF**	3.176*(2.5)	2.387*(1.3)	2.077*(1.2)	1.706(1.0)	2.555*(1.6)
**RUDOLPH**	8.741*(7.1)	7.263*(5.7)	2.811*(1.3)	1.799(0.95)	7.123*(5.8)
**VParNet**	1.350(0.28)	1.387(0.36)	1.736(1.0)	1.834(1.4)	1.366(0.32)
**AVD(%):**					
**FreeSurfer**	15.4*(5.5)	14.6*(7.2)	36.0(46)	13.9(8.1)	14.2*(6.8)
**MALPEM**	8.34(11)	7.46(9.9)	28.0(25)	9.52(7.0)	7.35(9.3)
**JLF**	16.9*(6.5)	14.1*(6.2)	25.1*(13)	7.04(6.6)	15.0*(6.0)
**RUDOLPH**	18.2(26)	17.7(27)	43.5*(42)	13.7(12)	16.3(22)
**VParNet**	10.1(5.2)	10.0(6.3)	13.7(11)	11.0(8.3)	9.72(5.6)
**NPH:**					
**DSC:**					
**FreeSurfer**	0.941*(0.03)	0.938*(0.03)	0.839*(0.06)	0.735*(0.08)	0.936*(0.03)
**MALPEM**	0.910*(0.12)	0.909*(0.12)	0.808*(0.13)	0.727*(0.08)	0.905*(0.11)
**JLF**	0.929*(0.05)	0.931*(0.05)	0.869*(0.06)	0.842*(0.05)	0.929*(0.05)
**RUDOLPH**	0.925*(0.07)	0.927*(0.07)	0.856*(0.09)	0.801*(0.08)	0.925*(0.07)
**VParNet**	0.974(0.02)	0.975(0.02)	0.917(0.07)	0.904(0.05)	0.973(0.02)
**95% HD:**					
**FreeSurfer**	1.810*(1.4)	1.874*(1.0)	2.383*(1.1)	7.034*(3.2)	2.418*(1.2)
**MALPEM**	3.067*(5.2)	3.000*(5.5)	2.784*(2.3)	9.654*(3.5)	3.969*(5.0)
**JLF**	2.156*(2.8)	2.136*(3.0)	1.794*(0.85)	2.140*(1.3)	3.121*(2.2)
**RUDOLPH**	2.761*(3.5)	2.570*(3.5)	1.870*(0.94)	2.346*(1.3)	3.335*(3.0)
**VParNet**	1.092(0.75)	1.030(0.49)	1.566(1.4)	1.560(1.1)	1.086(0.54)
**AVD(%):**					
**FreeSurfer**	5.37*(5.5)	5.78(7.4)	14.7(21)	54.5*(29)	5.98*(7.0)
**MALPEM**	15.2*(17)	15.4*(18)	27.1*(29)	71.0*(33)	16.1*(17)
**JLF**	11.9*(13)	11.9*(14)	16.8*(18)	20.7*(18)	11.5*(13)
**RUDOLPH**	16.4*(18)	16.2*(20)	26.9*(32)	39.7*(29)	16.4*(19)
**VParNet**	3.55(3.6)	3.47(3.7)	8.89(10)	10.1(9.3)	3.62(3.8)

i^{th} group as testing set and the remaining 4 groups as training set. Among the training set, 5 NMM images and 10 NPH images were used for validation. Quantitative evaluation of the 5 networks is shown in Table 2. We conducted a Mann–Whitney U test (Mann and Whitney, 1947) with α-level of 0.01 to compare each pair of the

5 networks. The differences between any two networks in terms of the three evaluation metrics are not significant except the AVD of the right lateral ventricle between TestFold-3 and TestFold-4, which has $p = 0.0051$.

In this experiment, we evaluated the performance of the proposed network trained on different partitions of the data. The 5 networks produced similar parcellation results in terms of DSC, 95% HD, and AVD, indicating that the proposed network performs well on unseen data.

Table 2: The mean (standard deviation) of Dice similarity coefficient (DSC), 95% Hausdorff distance (HD), and absolute volume difference (AVD) for each cross-validation group over 10 NMM data and 19 NPH data. TestFold-*i* means the ith group was taken as testing data and the network was trained on the remaining 4 groups.

	**RLV**	**LLV**	**3rd**	**4th**	**Whole**
**NMM:**					
**DSC:**					
**TestFold-1**	0.949(0.04)	0.951(0.04)	0.892(0.07)	0.889(0.05)	0.949(0.04)
**TestFold-2**	0.952(0.04)	0.951(0.04)	0.883(0.09)	0.889(0.05)	0.949(0.04)
**TestFold-3**	0.948(0.05)	0.949(0.05)	0.888(0.09)	0.881(0.06)	0.946(0.05)
**TestFold-4**	0.943(0.05)	0.948(0.04)	0.865(0.09)	0.890(0.04)	0.944(0.04)
**TestFold-5**	0.952(0.04)	0.953(0.04)	0.899(0.05)	0.897(0.04)	0.950(0.04)
**95% HD:**					
**TestFold-1**	1.013(0.24)	0.989(0.29)	1.397(0.70)	1.657(1.27)	1.211(0.84)
**TestFold-2**	1.075(0.42)	1.059(0.38)	1.312(0.64)	1.463(1.09)	1.097(0.44)
**TestFold-3**	1.087(0.60)	1.085(0.42)	1.288(0.81)	1.490(0.85)	1.111(0.40)
**TestFold-4**	1.734(2.57)	1.065(0.30)	1.868(1.59)	1.181(0.32)	1.301(0.61)
**TestFold-5**	1.071(0.45)	1.049(0.37)	1.317(0.79)	1.512(0.78)	1.050(0.30)
**AVD(%):**					
**TestFold-1**	5.12(4.4)	5.05(4.0)	9.11(7.9)	9.13(8.7)	4.69(3.4)
**TestFold-2**	4.31(4.1)	4.42(5.0)	11.4(12)	8.54(9.1)	4.36(4.7)
**TestFold-3**	3.97(5.8)	4.54(6.0)	9.83(11)	11.8(11)	4.11(5.7)
**TestFold-4**	6.35(5.2)	5.99(5.1)	12.8(12)	9.67(8.2)	6.10(5.0)
**TestFold-5**	4.60(4.1)	4.22(4.1)	8.57(7.5)	9.38(6.0)	4.27(3.5)

3. Effect of training data on VParNet

A preliminary version of this work was reported in conference form (Shao et al., 2018b), where we investigated the effect of training data for a deep network. We trained the same network (VParNet) three times with different training data: VParNet-1 using 13 T1-w MRIs from the NMM data set, VParNet-2 using 38 T1-w MRIs including the same 13 NMM MRIs in VParNet1 and additional 25 NMM MRIs, and VParNet-3 using 38 T1-w MRIs including the same 13 NMM MRIs in VParNet1 and 25 NPH MRIs. The training images were chosen to cover the entire spectrum of ventricle sizes in the NMM or the NPH data set. We augmented the training data by left/right flipping, random rotation, and elastic deformation. The batch size was 1. The leaky ReLU negative slope was 0.1 and the dropout rate was 0.2. The loss function was one minus the mean DSC of all the labels. The network was trained for 50 epochs each time using

Table 3: The mean (standard deviation) of Dice similarity coefficient (DSC), 95% Hausdorff distance (HD), and absolute volume difference (AVD) over 12 T1-w MRIs from the NMM data set and 70 T1-w MRIs from the NPH data set. The asterisk means the corresponding method (VParNet-1, VParNet-2) is significantly different (p-value < 0.01) from VParNet-3 in terms of the evaluation metric. The median of 95% HD of the 3rd ventricle on NPH data set from VParNet-2 is reported instead of the mean. The reason is that VParNet-2 failed to output 3rd ventricle label on 2 NPH testing images and the corresponding 95% HD was infinity.

	**RLV**	**LLV**	**3rd**	**4th**	**Whole**
**NMM:**					
**DSC:**					
**VParNet-1**	0.885*(0.04)	0.889(0.04)	0.812(0.04)	0.853(0.03)	0.888(0.03)
**VParNet-2**	0.892(0.03)	0.893(0.04)	0.820*(0.05)	0.860*(0.03)	0.890(0.03)
**VParNet-3**	0.893(0.04)	0.893(0.04)	0.806(0.05)	0.847(0.04)	0.889(0.04)
**95% HD:**					
**VParNet-1**	1.221(0.25)	1.276(0.29)	1.592(1.1)	1.655(0.63)	1.170(0.13)
**VParNet-2**	1.115(0.20)	1.203(0.44)	1.295*(0.79)	1.502(0.71)	1.182(0.22)
**VParNet-3**	1.076(0.13)	1.241(0.69)	1.344(0.69)	1.736(0.98)	1.198(0.25)
**AVD(%):**					
**VParNet-1**	9.60(7.1)	8.64(5.4)	16.3(12)	11.0(13)	8.51(5.4)
**VParNet-2**	7.44(4.4)	8.24(6.8)	10.7(6.9)	10.5(12)	6.96(5.0)
**VParNet-3**	8.07(6.4)	5.21(5.7)	17.6(12)	12.9(14)	6.58(5.3)
**NPH:**					
**DSC:**					
**VParNet-1**	0.839*(0.15)	0.832*(0.15)	0.727*(0.21)	0.787*(0.11)	0.833*(0.14)
**VParNet-2**	0.484*(0.25)	0.480*(0.25)	0.275*(0.28)	0.684*(0.18)	0.482*(0.24)
**VParNet-3**	0.972(0.02)	0.974(0.02)	0.916(0.05)	0.902(0.04)	0.972(0.02)
**95% HD:**					
**VParNet-1**	6.542*(7.1)	7.294*(7.2)	9.700*(16)	11.76*(8.8)	6.108*(4.8)
**VParNet-2**	15.29*(9.4)	17.23*(7.8)	21.19*	29.55*(28)	15.03*(7.4)
**VParNet-3**	1.731(3.8)	1.341(2.0)	1.689(1.3)	1.494(0.89)	1.533(1.7)
**AVD(%):**					
**VParNet-1**	20.8*(20)	21.9*(20)	31.0*(27)	20.9*(17)	21.7*(19)
**VParNet-2**	59.7*(26)	60.4*(26)	76.9*(28)	26.6*(22)	59.4*(27)
**VParNet-3**	3.26(3.9)	3.15(3.6)	8.60(7.5)	11.5(9.5)	3.29(3.8)

the Adam optimizer with learning rate 0.001, the exponential decay rate $\beta_1 = 0.9$, $\beta_2 = 0.999$, and $\epsilon = 10^{-7}$.

The remaining 12 NMM images and 70 NPH images formed the testing data set. The quantitative evaluation of the parcellation results generated by the three networks are presented in Table 3. A paired Wilcoxon signed-rank test with $\alpha = 0.01$ was conducted to compare VParNet-3 with VParNet-1 and VParNet-2 and the asterisks mark the significant difference.

We note that the three networks showed similar performance on the NMM testing data. However, VParNet-1 and VParNet-2 showed poor performance on the NPH testing data. VParNet-2 provided worse parcellation results than VParNet-1 despite having more training data. One possible explanation is that adding more NMM training data in VParNet-2 made the network overfit on the NMM data set. The experiments showed the importance of careful training data selection in deep neural networks. Although the

NMM data set contained some elderly subjects with enlarged ventricles, the two data sets were acquired under different scanning protocols and had different image contrasts. Therefore, the network trained on healthy NMM data cannot handle NPH cases.

References

Aaron Carass, Muhan Shao, Xiang Li, Blake E Dewey, Ari M Blitz, Snehashis Roy, Dzung L Pham, Jerry L Prince, and Lotta M Ellingsen. Whole brain parcellation with pathology: Validation on ventriculomegaly patients. In *International Workshop on Patch-Based Techniques in Medical Imaging*, pages 20–28. Springer, 2017.

Anders M Dale, Bruce Fischl, and Martin I Sereno. Cortical surface-based analysis: I. Segmentation and surface reconstruction. *NeuroImage*, 9(2):179–194, 1999.

Lotta M Ellingsen, Snehashis Roy, Aaron Carass, Ari M Blitz, Dzung L Pham, and Jerry L Prince. Segmentation and labeling of the ventricular system in normal pressure hydrocephalus using patch-based tissue classification and multi-atlas labeling. In *Medical Imaging 2016: Image Processing*, volume 9784, page 97840G. International Society for Optics and Photonics, 2016.

Bruce Fischl. FreeSurfer. *NeuroImage*, 62(2):774–781, 2012.

Bruce Fischl, David H Salat, Evelina Busa, Marilyn Albert, Megan Dieterich, Christian Haselgrove, Andre Van Der Kouwe, Ron Killiany, David Kennedy, Shuna Klaveness, et al. Whole brain segmentation: automated labeling of neuroanatomical structures in the human brain. *Neuron*, 33(3):341–355, 2002.

Christian Ledig, Rolf A Heckemann, Alexander Hammers, Juan Carlos Lopez, Virginia FJ Newcombe, Antonios Makropoulos, Jyrki Lötjönen, David K Menon, and Daniel Rueckert. Robust whole-brain segmentation: Application to traumatic brain injury. *Medical Image Analysis*, 21(1):40–58, Apr 2015.

Henry B Mann and Donald R Whitney. On a test of whether one of two random variables is stochastically larger than the other. *The annals of mathematical statistics*, pages 50–60, 1947.

Muhan Shao, Aaron Carass, Xiang Li, Blake E Dewey, Ari M Blitz, Jerry L Prince, and Lotta M Ellingsen. Multi-atlas segmentation of the hydrocephalus brain using an adaptive ventricle atlas. In *Medical Imaging 2018: Biomedical Applications in Molecular, Structural, and Functional Imaging*, volume 10578, page 105780F. International Society for Optics and Photonics, 2018a.

Muhan Shao, Shuo Han, Aaron Carass, Xiang Li, Ari M Blitz, Jerry L Prince, and Lotta M Ellingsen. Shortcomings of Ventricle Segmentation Using Deep Convolutional Networks. In *Understanding and Interpreting Machine Learning in Medical Image Computing Applications*, pages 79–86. Springer, 2018b.

Hongzhi Wang, Jung W Suh, Sandhitsu R Das, John B Pluta, Caryne Craige, and Paul A Yushkevich. Multi-atlas segmentation with joint label fusion. *IEEE Transactions on Pattern Analysis and Machine Intelligence*, 35(3):611–623, 2013.

Frank Wilcoxon. Individual comparisons by ranking methods. *Biometrics Bulletin*, 1 (6):80–83, 1945.
